# A Novel Amino Acid Composition Ameliorates Short-Term Muscle Disuse Atrophy in Healthy Young Men

**DOI:** 10.3389/fnut.2019.00105

**Published:** 2019-07-10

**Authors:** Tanya M. Holloway, Chris McGlory, Sean McKellar, Adrienne Morgan, Mike Hamill, Raffi Afeyan, William Comb, Scharmen Confer, Peng Zhao, Mark Hinton, Olga Kubassova, Manu V. Chakravarthy, Stuart M. Phillips

**Affiliations:** ^1^Department of Kinesiology, McMaster University, Hamilton, ON, Canada; ^2^Axcella Health, Inc., Cambridge, MA, United States; ^3^Image Analysis Group, Philadelphia, PA, United States

**Keywords:** skeletal muscle, leucine, protein turnover, strength, function, recovery

## Abstract

Skeletal muscle disuse leads to atrophy, declines in muscle function, and metabolic dysfunction that are often slow to recover. Strategies to mitigate these effects would be clinically relevant. In a double-blind randomized-controlled pilot trial, we examined the safety and tolerability as well as the atrophy mitigating effect of a novel amino acid composition (AXA2678), during single limb immobilization. Twenty healthy young men were randomly assigned (10 per group) to receive AXA2678 or an excipient- and energy-matched non-amino acid containing placebo (PL) for 28d: days 1–7, pre-immobilization; days 8–15, immobilization; and days 16–28 post-immobilization recovery. Muscle biopsies were taken on d1, d8 (immobilization start), d15 (immobilization end), and d28 (post-immobilization recovery). Magnetic resonance imaging (MRI) was utilized to assess quadriceps muscle volume (Mvol), muscle cross-sectional area (CSA), and muscle fat-fraction (FF: the fraction of muscle occupied by fat). Maximal voluntary leg isometric torque was assessed by dynamometry. Administration of AXA2678 attenuated muscle disuse atrophy compared to PL (*p* < 0.05) with changes from d8 to d15 in PL: ΔMvol = −2.4 ± 2.3% and ΔCSA = −3.1% ± 2.1%, both *p* < 0.001 vs. zero; against AXA2678: ΔMvol: −0.7 ± 1.8% and ΔCSA: −0.7 ± 2.1%, both *p* > 0.3 vs. zero; and *p* < 0.05 between treatment conditions for CSA. During immobilization, muscle FF increased in PL but not in AXA2678 (PL: 12.8 ± 6.1%, AXA2678: 0.4 ± 3.1%; *p* < 0.05). Immobilization resulted in similar reductions in peak leg isometric torque and change in time-to-peak (TTP) torque in both groups. Recovery (d15–d28) of peak torque and TTP torque was also not different between groups, but showed a trend for better recovery in the AXA2678 group. Thrice daily consumption of AXA2678 for 28d was found to be safe and well-tolerated. Additionally, AXA2678 attenuated atrophy, and attenuated accumulation of fat during short-term disuse. Further investigations on the administration of AXA2678 in conditions of muscle disuse are warranted.

**Clinical Trial Registration:** https://clinicaltrials.gov, identifier: NCT03267745.

## Introduction

Skeletal muscle atrophy and strength loss are rapid during limb disuse (1) and bed rest (2). Oftentimes, disuse-induced atrophy does not demonstrate complete recovery despite returning to normal activity (3) or even with the potent stimulus of resistance exercise (4). Interventions to mitigate disuse-induced atrophy and function and accelerate recovery are of clinical relevance (5–7).

The anti-atrophic effectiveness of amino acid- or protein-based nutritional interventions during disuse atrophy have been inconsistent, with some studies demonstrating benefit (8–11), while others have not (12, 13). A reproducible feature of muscle disuse in humans is a decline in the rates of fasted- and fed-state muscle protein synthesis (MPS) (14–17), which has been coined anabolic resistance (15, 18). It appears clear that in humans the decline in fasted MPS can account for the majority of the loss of muscle mass with uncomplicated (i.e., non-pathologic) muscle disuse (19, 20); hence, the decline in MPS should be targeted in mitigating disuse atrophy. Given its role in stimulating MPS, leucine would appear to be an important EAA (21) to include in any formulation designed to mitigate disuse atrophy. Nonetheless, provision of protein (12) has been shown to be ineffective, leucine monotherapy to attenuate disuse atrophy has also yielded equivocal results showing attenuation (11) or no effect (13) on disuse-induced atrophy and functional declines. In contrast, atrophy during bed rest was effectively counteracted with higher doses of EAA plus carbohydrate (8, 9). It may also be that dietary control might be important in distinguishing studies that have not shown an effect of protein or amino acid supplementation (12, 13) vs. those that have (8, 9, 11).

Disuse atrophy is multifactorial process that involves a number of processes linked to muscle protein turnover including muscle microvascular perfusion (7), intrinsic anabolic resistance to EAA provision (7, 20), and oxidative stress (22, 23). We propose that it is a confluence of these factors that gives rise to the atrophy phenotype. Thus, beyond EAA, certain non-essential amino acids (NEAA) could play important roles in mitigating disuse atrophy. These NEAA include glutamine which may function by attenuating catabolism (24). The NEAA arginine may serve to maintain capillary perfusion (25) and thus delivery of substrate or directly stimulate MPS (26). Oxidative stress has been postulated to be a key process in disuse atrophy (22) and thus preservation of thiol groups in their reduced state via the provision of n-acetylcysteine (NAC) may also aid in mitigating atrophy (27).

The novel amino acid formulation AXA2678 contains select EAA (leucine, isoleucine, valine, lysine, histidine, phenylalanine, and threonine) and NEAA (arginine, glutamine, and NAC) that target a number of potential disuse-induced sequelae that could contribute to muscle atrophy. In this investigation, we aimed to assess the safety and tolerability of AXA2678 in young men across 28d of supplementation that included a period of disuse. We also assess the impact of AXA2678 on muscle cross-sectional area, muscle volume and muscle function. We hypothesized that thrice daily administration AXA2678 would be safe, well-tolerated and would mitigate muscle atrophy in healthy young men undergoing 7d of unilateral limb immobilization.

## Methods

### Participants

This study was carried out in accordance with the recommendations of the Canadian Tri-Council policy statement on the use of human participants in research (http://www.pre.ethics.gc.ca/pdf/eng/tcps2-2014/TCPS_2_FINAL_Web.pdf) with written informed consent from all subjects. All subjects gave written informed consent in accordance with the Declaration of Helsinki. The protocol was reviewed and approved by the Hamilton Integrated Research Ethics Board (HiREB, Protocol 3365). This trial was registered (https://clinicaltrials.gov) as NCT03267745.

### Protocol

We conducted a double-blind parallel group pilot trial in which subjects were randomly assigned (computer-generated sequence) to receive either a proprietary amino acid-containing formulation (AXA2678) or an excipient- and energy-matched placebo (maltodextrin) liquid drink thrice daily. The composition of amino acids within AXA2678 is shown in Table 1. Subjects consumed 23.7 g of either AXA2678 or placebo thrice daily 2 h after breakfast, lunch, and dinner for 28 consecutive days. The primary outcomes were safety and tolerability. We also assessed the impact of the formulation on muscle atrophy.

**Table 1:** Amino acid composition of AXA2678 per dose.

**Amino acid**	**Dose (g)**
Leucine	4.00
Isoleucine	2.00
Valine	2.00
Arginine^a^	7.24
Glutamine	5.32
Lysine^b^	1.40
Histidine	0.32
Phenylalanine	0.32
Threonine	0.68
NAC	0.60

a *As hydrochloride salt*.

b *As acetate salt*.

*NAC, n-acetylcysteine*.

**Figure 1:**
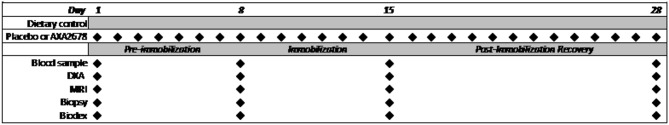
Schematic of study protocol showing the periods of the study and timing of data collection.

A schematic timeline of the study protocol and measurements is shown in Figure 1. The 28d protocol included a 7d run-in phase of administration of AXA2678 or PL prior to the unilateral knee immobilization (pre-immobilization), 7d of immobilization and 14d of post-immobilization recovery. During the recovery period dietary control was continued and subjects were instructed to resume their habitual daily routines (“passive” recovery). Daily activity was monitored with a SenseWear Pro Armband™ (Body Media, Pittsburgh, PA) as previously described (3).

### Dietary Intake

Dietary intake was standardized using previously published procedures (28, 29) for the duration of the study by providing subjects all meals in addition to pre-mixed drinks (e.g., placebo or AXA2678). Briefly, participants were provided with all meals and beverages to consume throughout the intervention period (with the exception of water and non-caloric drinks, which were *ad libitum*). Diets were designed to achieve energy balance and, based on personal-preference, meal plans were derived with protein from dietary sources held constant at 1.0 g/kg/day. Compliance with the nutritional intervention (i.e., consumption of all the provided study meals, including placebo, or AXA2678 supplementation) was assessed by daily contact with participants, returned drink pouches, and food diaries. Compliance with meal consumption and supplement ingestion was similar between groups and exceeded 95% in all subjects in both groups.

### Immobilization

Participants underwent 7 d (days 8–15; Figure 1) of single leg immobilization by means of a knee brace, described previously (15, 17), allowing for the contralateral limb to act as an internal control. The immobilized leg was chosen as the dominant leg based on the peak torque during an isometric maximal voluntary contractions (MVC) from the Biodex. The brace was placed in a fixed flexion position 40° from full extension (i.e., 140°) with a plastic strap in place to prevent tampering with the brace. At this angle, the brace allowed for full clearance of the subjects' toe from the ground while moving using crutches. It also notable that at this angle the subject could not place significant downward force on their foot to effectively activate their knee extensors. The brace was removed to check for pressure points daily and resealed with a custom-modified plastic strap that was melted to seal the strap and to prevent removal of the brace. Previous use of this model has, in our hands, proved effective and has resulted in degrees of muscle atrophy on par with that seen with casting and short-term bed rest (15, 17, 30–32).

### Knee Extensor Isometric Torque and Time-to-Peak (TTP) Torque

Isometric knee extensor torque was measured with the use of a Biodex dynamometer (Shirley, NY) as previously reported (33). Briefly, sessions consisted of three 5s unilateral knee extension maximum voluntary contractions (MVC) with 1 min of rest between contractions. The analog torque signal for the Biodex was sampled at 2,000 Hz with PowerLab 3 data acquisition system (ADInstruments, Bella Vista, Australia). The knee angle was set at 120° and the highest recorded torque (best of three attempts) for the leg was taken as the MVC peak torque. The TTP torque was taken as the time from the first rise in force to the time to achieve peak torque. Onset of contraction was defined as the instant when force production exceeded the baseline level force by 5% of the maximal force value. Any trials with a visible initial countermovement were discarded (34).

### Dual Energy X-Ray Absorptiometry (DEXA)

DEXA measurements were performed using a GE Lunar iDXA total body scanner (GE Medical Systems Lunar, Madison, WI) and analyzed with software (Lunar enCORE version 14.1; GE Medical Systems) in the medium scan mode. A three-compartment Universal Whole Body DXA Phantom: Oscar, Jr. (Orthometrix, Naples, FL) was utilized as a calibration standard for the equipment on each experimental day. Standard analysis regions (head, torso, arms, and legs) were subdivided by the software and manually verified by a third-party investigator who was blinded to the treatments.

### Magnetic Resonance Imaging (MRI)

MRI was used to capture axial (transverse) images were obtained from both thighs from the distal end of femur to greater trochanter as previously described at day 0, day 8, day 15, and day 28. A fast-recovery, fast spin echo pulse sequence was used, along with IDEAL (iterative decomposition of water and fat with echo asymmetry and least-squares estimation) post-processing to obtain water-only, fat-only, in-phase (IP), and out-of-phase (OP) images of the thighs using a GE high fidelity 3T magnet. The following parameters were employed: TR = 2,000 ms, TE = 30 ms, refocusing flip angle = 111 degrees, echo train length = 6, ASSET (parallel imaging factor) = 2, field of view = 42 × 21 cm, acquisition matrix = 512 × 256, 3 mm slice thickness, 0 mm slice gap, a total of ~160 slices were acquired, depending on length of the thigh. The scans were uploaded onto AnalyzePro software (Overland Park, KS) and the 50% region between the greater trochanter of the hip and lateral epicondyle of the knee were used for analysis. The segmentation features of the software were used to differentiate between regions of interests (ROI) for bone, fat, and muscle in each leg. Then every third slice in the 50% region was manually traced for the quadriceps muscles of both legs. The software was then able to take every third slice that was manually measured and calculated that data for every slice in the 50% region to get an estimate of quadriceps volume. Muscle CSA was expressed in mm^2^ and muscle volume in mm^3^.

### Muscular Fat Fraction (FF)

The FF of each muscle scan (Supplemental Figure 2) was assessed in a blinded fashion by analysts with the Image Analysis Group (IAG; Phildelphia, PA). Briefly, in-phase and out-of-phase images (35) from the MRI scans were uploaded into DYNAMIKA (IAG, Image Analysis Group, Philadephia, PA) software and automatically processed on a pixel-by-pixel basis. Specifically, pure water only and fat only maps were derived using the following formulae:

12 [IP + OP]=12[(W + F)+(W - F)]=12[2W]                            =W(water only)12 [IP - OP]=12[(W + F)-(W - F)]=12[2F]                            =F(fat only)

Where IP is the signal intensity of pixels from the in-phase image and OP is the signal intensity of pixels from the out-of-phase image (35). W and F are the signal contributions from water and fat (35). The FF, inside the boundaries of each muscle, were estimated as FF = F/ (W + F). Once the segmentation was carried out on all image slices, the segmented per-slice ROI were grouped into a volume and FF calculated for the entire leg segment.

### Muscle Biopsy

Percutaneous needle biopsies (5 mm Bergstrom needle customized for manual suction) were obtained from the medial portion of the *vastus lateralis* (~10–15 cm above the patella) under fasting conditions and after administration of local anesthesia (1% lidocaine). Biopsies were taken at baseline (d1), d8 (pre-immobilization), d15 (post-immobilization), and d28 (recovery). Biopsy samples were embedded in optimal cutting temperature compound (OCT), frozen in liquid nitrogen-cooled isopentane (Sigma, St. Louis, MO) and stored at −80°C for further immunohistochemical analysis.

### Fiber-Type Specific Cross-Sectional Area

Biopsy samples previously mounted in OCT underwent immunohistochemical staining to determine muscle and fiber-type specific cross-sectional area. Tissue was sectioned using a cryostat set to −20°C and the 5 μm thick cryosections from each time point (d1, d8, d15, d28) from a given subject were mounted on the same glass slide. To measure fiber type specific CSA, slides were retrieved from the −80°C freezer and thawed at room temperature for 30 min. Samples were treated and analyzed as described previously for fiber type and size (36, 37).

### Venous Blood Sampling

Blood samples were taken under fasting conditions on days 1, 8, 15, and 28 from an antecubital vein and plasma samples were analyzed at ICON central laboratories (Farmingdale, NY) using standard laboratory methods (CV for all methods <5%). Safety was assessed with a liver function panel [alanine aminotransferase (ALT), albumin, alkaline phosphatase (ALP), aspartate aminotransferase (AST), bicarbonate, bilirubin, blood urea nitrogen (BUN), creatinine, and total protein].

### Statistics

Data were analyzed using two-way repeated measures analysis of variance (ANOVA) to compare the PL and AXA2678 groups across time. Where significant F ratios were observed *t*-tests were used to examine pairwise differences. We also examined changes in variables for clarity of discrimination between conditions. Single point in time changes were analyzed using non-paired *t*-tests of *t*-tests of the mean vs. zero. Statistical significance was accepted when *p* ≤ 0.05. Statistical analyses were performed using SAS V9.4 (SAS Institute, Cary, NC). Graphical results are presented as means±SD or as box and-whisker plots including the median (line), mean (+) with interquartile range (boxes), and range (whiskers indicating minimum and maximum values). Tabular data are presented as means±SD.

## Results

### Participant Characteristics, Activity Level, and Dietary Intake

Twenty young men consented and completed the study (AA: *n* = 10; PL: *n* = 10; Supplemental Figure 1). One subject in the PL group was excluded from analysis as we suspected the brace had been removed. Their SenseWear™ data indicated that the very little reduction in daily steps during the immobilization phase. On exit from the study, 8 of 19 subjects correctly identified their group assignment. Mean ages and body weights were similar at baseline (Table 2). All participants reported wearing the brace continuously for 7 days, with the exception of one participant who required brace adjustment for comfort on day 3 of the immobilization period. During the 7 days of immobilization (day 8–15) body weight was maintained in both groups and no differences in body composition were observed by DEXA (data not shown).

**Table 2:** Participants' body weight, fat mass (FM), fat- and bone-free mass (FBFM), daily steps, and energy intake during the study protocol.

	**PL**			**AXA2678**		
	**d1**	**d8**	**d28**	**d1**	**d8**	**d28**
Weight (kg)	81.7 ± 6.1	81.4 ± 6.1	81.5 ± 6.0	81.9 ± 4.7	81.7 ± 4.8	81.2 ± 4.6
FM (kg)	16.0 ± 3.7	16.8 ± 3.8	16.9 ± 3.9	15.9 ± 8.5	15.2 ± 9.2	15.4 ± 8.5
FBFM (kg)	59.4 ± 2.6	59.8 ± 3.2	59.0 ± 3.1	60.6 ± 7.9	60.6 ± 7.4	60.8 ± 7.6
Steps (per d)	10427 ± 1068	5231 ± 595^*^	8431 ± 911	9385 ± 1193	3363 ± 913^*^^†^	7571 ± 939
Energy (kcal/d)	2759 ± 260	2521 ± 253^*^	2855 ± 291	2653 ± 260	2462 ± 220^*^	2633 ± 249

*Pre (pre-immobilization: days 1–7), Immob (immobilization: days 8–15), and Post (post-immobilization [recovery]: days 16–28). Energy intake in kilocalories (kcal); %CHO, percentage of dietary energy from carbohydrate; %Fat, percentage of dietary energy from fat; and %Pro, percentage of dietary energy from protein. Data are expressed as means ± SD*.

* *Different from other time points in that group (p < 0.001)*.

† *Difference between PL and AXA2678, at the same time point (p < 0.05)*.

As expected, activity (steps/day) substantially decreased with limb immobilization (Table 2). However, the decrease in daily steps was significantly greater in the AXA2678 group compared to placebo (−68 vs. −55% from pre-immobilization steps, respectively; *p* < 0.05). At the end of the recovery period, activity in both groups had returned to pre-immobilization levels, with no significant differences between the two groups.

Dietary energy intake was lower, by design, during the immobilization period (Table 2). Dietary protein intake remained constant at 1.0 g protein/kg/d with an additional 71.1 g of AXA2678 daily in that group.

### Safety and Tolerability

No clinically meaningful changes in chemistry, hematology, or vital signs were observed with ingestion of AXA2678. Clinical chemistry was unremarkable with all values within normal ranges (Table 3). AXA2678 amounts and regimen followed in the current study were well-tolerated. Two non-serious adverse events (AE) of sore throat were reported in one subject taking AXA2678 on two separate occasions (study days 6 and 22) and deemed not related to AXA2678. Other non-serious AE included gas and nausea, which were reported simultaneously in one subject taking AXA2678 on study day 12 and were noted as possibly-related to AXA2678 administration. All AE were considered mild, resolved in within a day without intervention, and did not interrupt nor cause cessation of consumption of AXA2678.

**Table 3:** Blood clinical chemistry at study entry (d1) and exit (d28).

	**PL**		**AXA 2678**	
	**d1**	**d28**	**d1**	**d28**
ALT (U/L)	20.6 ± 11.4	19.3 ± 8.9	27.6 ± 12.5	23.3 ± 7.9
Albumin (g/dL)	4.8 ± 0.2	4.6 ± 0.2	4.8 ± 0.2	4.6 ± 0.2
ALP (U/L)	76.3 ± 12.8	79.0 ± 12.1	90.1 ± 24.7	89.3 ± 22.3
AST (U/L)	23.2 ± 9.2	20.4 ± 7.1	23.1 ± 2.5	21.0 ± 2.2
Bicarbonate (mEq/dL)	22.9 ± 2.3	23.0 ± 1.7	22.6 ± 1.5	23.4 ± 1.9
Bilirubin (mg/dL)	0.8 ± 0.3	0.8 ± 0.4	1.0 ± 0.7	0.9 ± 0.5
BUN (mg/dL)	17.5 ± 3.5	10.8 ± 2.6	17.7 ± 3.4	15.5 ± 3.9
Creatinine (mg/dL)	0.9 ± 0.1	0.9 ± 0.1	1.0 ± 0.1	1.0 ± 0.1
Protein (g/dL)	7.6 ± 0.4	7.2 ± 0.4	7.7 ± 0.5	7.3 ± 0.4

*Study entry, d1 (see Figure 1) and d28 (last study day)*.

*ALT, alanine aminotransferase; ALP, alkaline phosphatase; AST, aspartate aminotransferase; BUN, blood urea nitrogen. Data are expressed as means ± SD*.

### Quadriceps CSA, Muscle Volume, and FF by MRI

**Figure 2:**
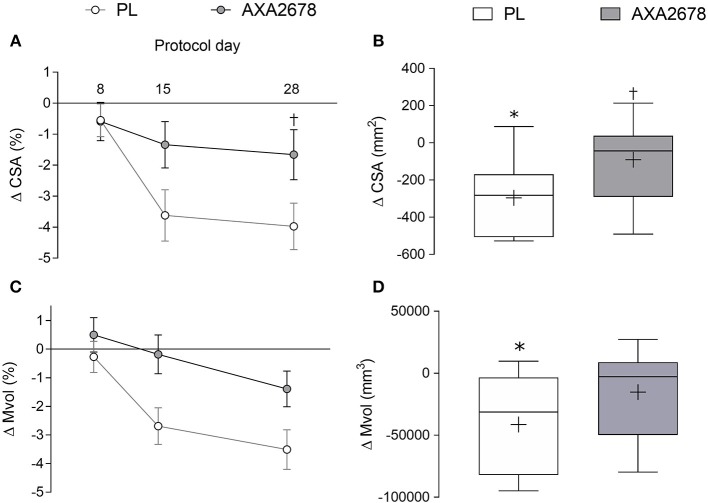
Relative and absolute changes in quadriceps muscle CSA [relative to day 1, panel **(A)**] and absolute changes during immobilization [day 15 min day 8, **(B)**]. Relative and absolute changes in quadriceps muscle volume [Mvol; relative to day 1, panel **(C)**] and absolute changes during immobilization [day 15 min day 8, panel **(D)**]. Data are presented as means ± SD **(A,C)**. Data are presented with interquartile range (boxes) ± range (minimum and maximum), where + indicates the group mean and the median is indicated by the lines **(B,D)**. ANOVA results panel A: time, *p* < 0.001; treatment, *p* = 0.06; time × treatment, *p* = 0.03. ANOVA results **(C)** time, *p* < 0.005; treatment, *p* = 0.03; time x treatment, *p* = 0.19. ^*^Significantly different from zero (*p* < 0.01). ^†^Signficantly different from PL at the same timepoint (*p* < 0.05).

The change in peak muscle CSA (the same slice location pre- and post-immobilization) and Mvol from the acquired MRI sequences is shown in Figure 2. In the PL group, quadriceps peak CSA, and Mvol declined significantly during immobilization (d8 to d15 peak CSA: −3.1% ± 2.1%, *p* < 0.001; Mvol: −2.4 ± 2.3%, *p* < 0.001; Figures 2A,C). In contrast, peak CSA, and Mvol did not decline with AXA2678 administration (d8 to d15: CSA: −0.7 ± 2.1%; Mvol: −0.7 ± 1.8%; both *p* > 0.3; Figures 2A,C). At both d15 and d28 the decline in muscle CSA attenuated in AXA2678 versus PL (Figure 2A; *p* < 0.05). The absolute change in CSA (Figure 2B) and Mvol (Figure 2D) during immobilization were signficantly different from zero in the PL group but not in the AXA2678 group (Figures 2B,D; *p* < 0.05) and the change in CSA was signficantly lower in AXA2678 than PL (Figure 2B; *p* < 0.05).

**Figure 3:**
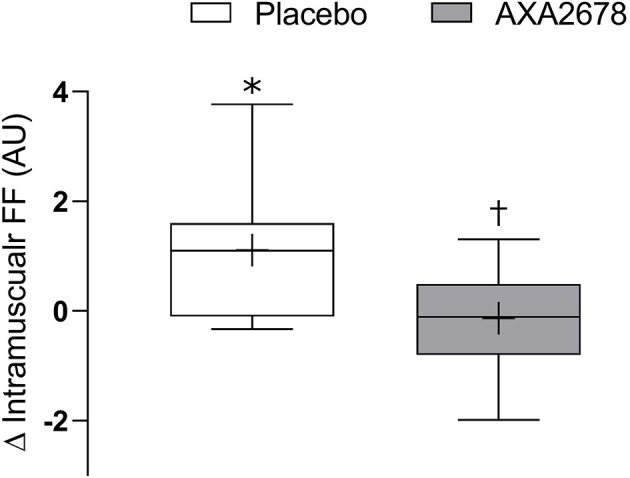
Change in muscle fat fraction (FF) during immobilization (d8–d15). Data are presented as median (lines) with interquartile range (boxes) ± range (minimum and maximum), where + indicates the group mean. *Signficantly different from zero (*p* < 0.05). ^†^signficantly different from PL (*p* < 0.05).

The FF of the muscle increased with immobilization in the PL group (1.1 ± 1.3 arbitrary units, 12.8 ± 6.1%, *p* < 0.05) but did not in the AXA2678 group (0.1 ± 0.7 arbitrary units, 0.4 ± 3.1%, *p* > 0.28) with a signficant difference noted between treatment groups (*p* < 0.05; Figure 3).

### Muscle Fiber Characteristics

Immunohistochemical analysis of muscle fibers did not reveal baseline differences in fiber CSA or fiber type distribution between PL and AXA2678 (data not shown). Immobilization did not result in a significant reduction of fiber CSA in either type I or type II fibers in either group (*p* > 0.2; data not shown). Immobilization resulted in a trend for a reduction in the percentage of type I fibers (*p* = 0.06) and a corresponding increase in type II fibers (*p* = 0.06) in PL, but not in the AXA2678 group (data not shown).

### Muscle Peak Isometric Torque and TTP Torque

**Figure 4:**
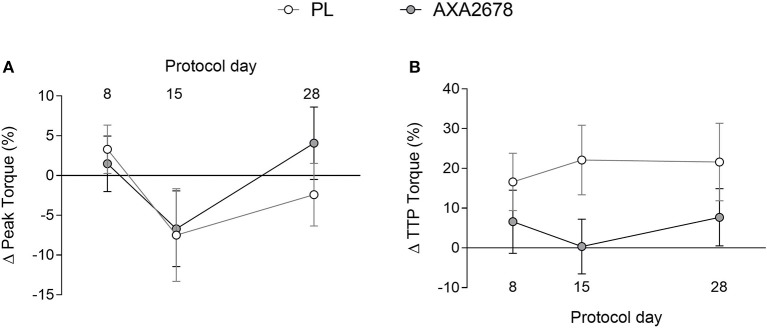
Relative changes in peak torque **(A)** and time to peak torque **(B)**. Values are presented as means ± SD. ANOVA **(A)** time, *p* = 0.15; treatment, *p* = 0.33; time × treatment, *p* = 0.7. ANOVA, **(B)** time, *p* = 0.8; treatment, *p* = 0.09; time x treatment, *p* = 0.38.

At baseline, no differences were observed between groups in peak knee extensor isometric torque or time to peak torque (data not shown). Immobilization resulted in similar relative declines in peak torque in both groups (Figure 4A). In addition, the TTP torque, while variable, showed no changes over time, or between groups (Figure 4B).

## Discussion

We report that AXA2678 administration over a 28d period, that included 7d of unilateral knee immobilization, was safe and well-tolerated in healthy young men. Furthermore, AXA2678 administration resulted in a preservation of quadriceps muscle volume, CSA, and an attenuation of muscle FF during immobilization. The declines in muscle CSA (−3.1%) and Mvol (−2.4%) in the PL group seen with 7d of immobilization are consistent with previous studies employing unilateral models of bracing or casting for immobilization (1, 12, 15, 17, 30, 31). Our results show that AXA2678 administration was safe, well-tolerated, attenuated short-term disuse atrophy, and ablated the rise in muscle FF during 7d of disuse, compared to a placebo.

Successive periods of short-term muscle disuse have negative health consequences even in young healthy subjects, including reduced insulin sensitivity (3, 38) and reduced muscle strength (1, 38). Importantly, recovery from disuse is oftentimes incomplete even with aggressive resistance training during rehabilitation (4). Furthermore, losses in Mvol and muscle CSA with only 7d of immobilization that we observed here (Figure 2) and that others have observed in similar or longer periods (1, 12, 13) are on par with what is seen with ~8–10 week of resistance training in younger persons (39). Thus, it takes ~4–5 times longer to gain muscle with loading than it does to lose it with unloading (40), which demonstrates that strategies to slow disuse atrophy are of paramount importance particularly in disuse situations (41).

Work from our group (15, 17), and others (14, 16), have attributed the loss of muscle mass with immobilization predominantly to reductions in both fasted-state and fed-state MPS (anabolic resistance); for review see (19, 20). Targeting disuse-induced anabolic resistance would thus require a therapy aimed at stimulating MPS (7). The provision of a moderate amount of protein (~21 or ~2 g of leucine twice daily) did not offset atrophy in older men (12). Amino acid supplementation may represent an effective strategy to combat anabolic resistance and thus, disuse atrophy (7). Leucine is a potent stimulator of MPS (21) and ~4 g of leucine per meal (0.06 g/kg/meal) has been shown to have some protective effect during disuse (11) although we acknowledge this is a disparate observation (13) who provided 2.5g leucine thrice daily. We observed potent effects of leucine-enriched protein supplementation on short-term (42) and longer-term MPS (43, 44). Therefore, we hypothesized that AXA2678, which when consumed three times daily, providing 12g of leucine, would aid in reducing atrophy in immobilization. Varying doses of EAA that included leucine (often in combination with carbohydrate) have been shown to be effective in mitigating muscle functional losses and losses of muscle mass in bed rest (8, 9).

The composition of AXA2678 is unique in that a number of constituent amino acids (arginine and glutamine) and n-acetylcysteine (NAC) have not been included in previous EAA-containing formulations designed to ameliorate atrophy (8–10). Thus, we have no direct comparison of AXA2678 to EAA-only containing formulations; however, we hypothesized that the constitution of AXA2678 may confer unique mechanistic advantages over leucine alone, leucine-enriched protein and potentially EAA. For example, NAC as an antioxidant for thiol groups that may be important in attenuating oxidative stress-induced atrophy pathways including proteolytic activation and depression of protein synthesis, for review see (23). There is some experimental support for the thesis that NAC is beneficial in mitigation from myopathies from pre-clinical models (27). In addition, the inclusion of arginine may play roles in attenuating atrophy by enhancing activation of the mechanistic target of rapamycin (26) or by enhancing capillary blood flow during disuse via increased nitric oxide biosynthesis (25). Another constituent of AXA2678 was glutamine, which may also have anti-catabolic applications particularly in disuse situations (24), which could be important in the early stages of disuse where proteolysis appears to be elevated (45). Nonetheless, we acknowledge that the proposed roles of any individual constituent NEAA in AXA2678 remains speculative at this point.

An interesting observation was that ingestion of AXA2678 ablated the small but significant increase in FF seen in the PL group with immobilization (Figure 3). The lipid identified on the MRI scan was intramuscular and as such our data are in contrast to other work reporting that short-term disuse is not accompanied by increased intramuscular lipid content (38, 46). However, the latter studies (38, 46) determined muscle fat content using biochemical methods from biopsies. It may be that small changes in intramuscular fat content during immobilization (47), are only detectable when summed across the whole muscle volume and not when analyzing a small portion of the muscle as would be the case in a biopsy (38, 46). The significance of the observation that AXA2678 prevented a rise in FF during disuse remains to be determined; however, we propose that attenuation in accumulation of muscle fat with disuse by AXA2678 could be clinically relevant as intramuscular fat accumulation is thought to contribute to metabolic dysregulation, including insulin resistance (48).

The loss of muscle mass in the current study was accompanied by a reduction in isometric torque in both PL and AXA2678 (Figure 4A), which was not significantly different between groups. Periodic muscle loading (resistance exercise) (31, 49) or neuromuscular electrical stimulation (50) is protective against losses of muscle mass. Some studies have shown mitigation of functional losses during bed rest that was 28d in duration (8) or leucine alone during 14d of bed rest (11). However, we note that other studies have not observed an effect on muscle function with protein ingestion (~16 g of EAA per day) during 5d of limb immobilization in older men (12) nor of leucine supplementation (2.5 g thrice daily) during 7d of single leg immobilization (13). We did not observe any mitigation of function in this relatively short-term study and we speculate that the duration of disuse may have to be extended to see such an effect.

We elected to use a placebo that was excipient-matched and identical in taste and color to AXA2678 as well as being isoenergetic, but containing only carbohydrate. Thus, the AXA2678 group was ingesting, vs. the PL group, an additional ~66.8 g of amino acids per day, of which ~46% were EAA (Table 1). This would represent ~66 g of more of a high quality protein such as milk protein [assuming ~50% by content EAA (51)] per day ingested by the AXA2678 group. Thus, our groups were not consuming isonitrogenous diets. Our design is similar in approach to other studies (EAA with/without carbohydrate or protein vs. a non-energetic or isoenergetic carbohydrate placebo) with broadly similar aims; nonetheless, as we have noted, these studies have shown divergent outcomes: a positive impact on mitigation of disuse atrophy with EAA and/or leucine supplementation (8–11), or no such effects (12, 13). We note that Dirks et al. (12) showed that in older men consuming an additional 35 g of high quality (milk-based) protein per day (representing an increase from 1.1 to 1.6 g protein/kg/d) afforded no protection against short-term disuse atrophy. While admittedly a different model, we note also that an additional 60 g of whey protein consumed during a 14d period of inactivity (not complete disuse) was not effective in mitigating muscle mass loss, but did enhance recovery (52). Future studies with AXA2678 could consider the role of EAA and/or NEAA and their contribution to the effects in disuse by using NEAA to create an isonitrogenous placebo against which to compare an active formulation.

In conclusion, thrice daily administration of AXA2678 was safe and well-tolerated. AXA2678 preserved muscle mass and attenuated intramuscular lipid accumulation during short-term muscle disuse-induced atrophy in young, healthy men. Future work could test this formulation in compromised populations, such as the elderly, for whom periodic disuse and inactivity are problematic due to incomplete recovery from such events (41).

## Data Availability

All datasets generated for this study are included in the manuscript/Supplementary Files.

## Author Contributions

TH, MC, SC, MiH, RA, WC, and SP were responsible for study design. TH, CM, AM, SM, and SP conducted the study. TH, CM, AM, SM, MaH, OK, PZ, and SP performed analysis of data. TH, MC, and SP drafted the paper. All authors are contributed to and approved the final version of the paper.

### Conflict of Interest Statement

MiH, RA, WC, SC, PZ, and MC are all employees of Axcella Health Inc. MaH and OK are employees of Image Analysis Group. SP declares that he has received travel expenses and consulting fees from Axcella Health, Inc. The remaining authors declare that the research was conducted in the absence of any commercial or financial relationships that could be construed as a potential conflict of interest.
