# Supplementary material for: A Novel Amino Acid Composition Ameliorates Short-Term Muscle Disuse Atrophy in Healthy Young Men
Source: Front Nutr. 2019 Jul 10;6:105. doi: 10.3389/fnut.2019.00105 (PMC6636393; doi:10.3389/fnut.2019.00105)

Supplementary material

Supplementary Figure 1. CONSORT Diagram.

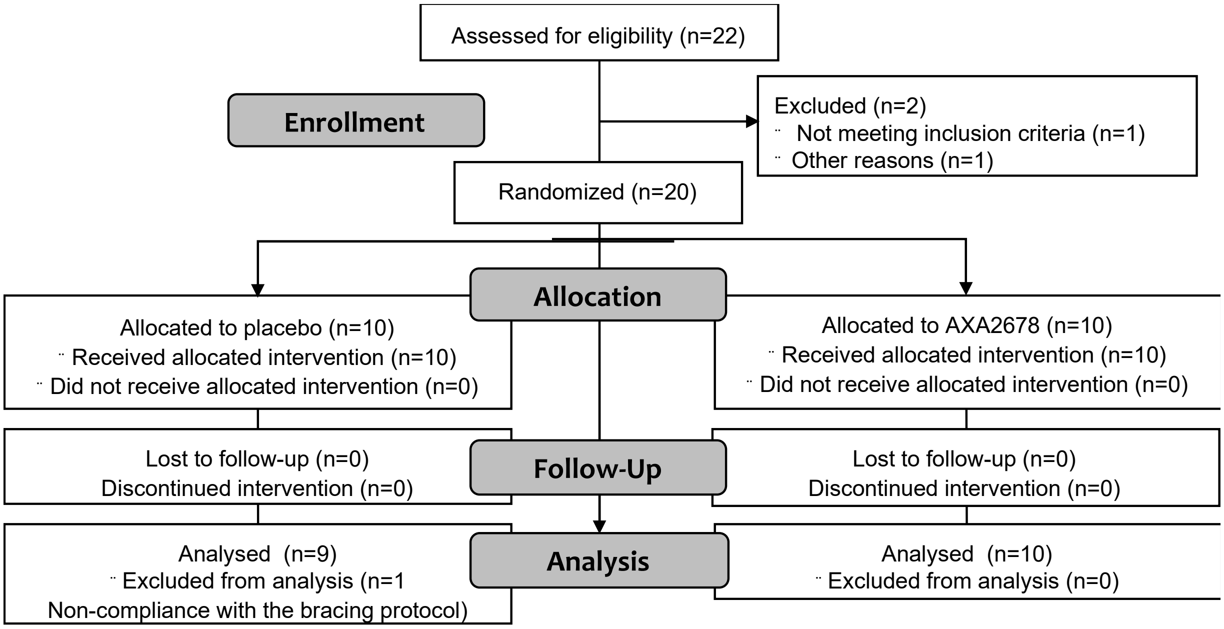

Supplementary Figure 2. A representative colour map showing variations in fat fraction on the scale from 0 to 1, with 1 indicating pixels located within fat and 0 – pixels located in muscle. In MRI scans, the muscle is generally dark except where infiltrated by fat.

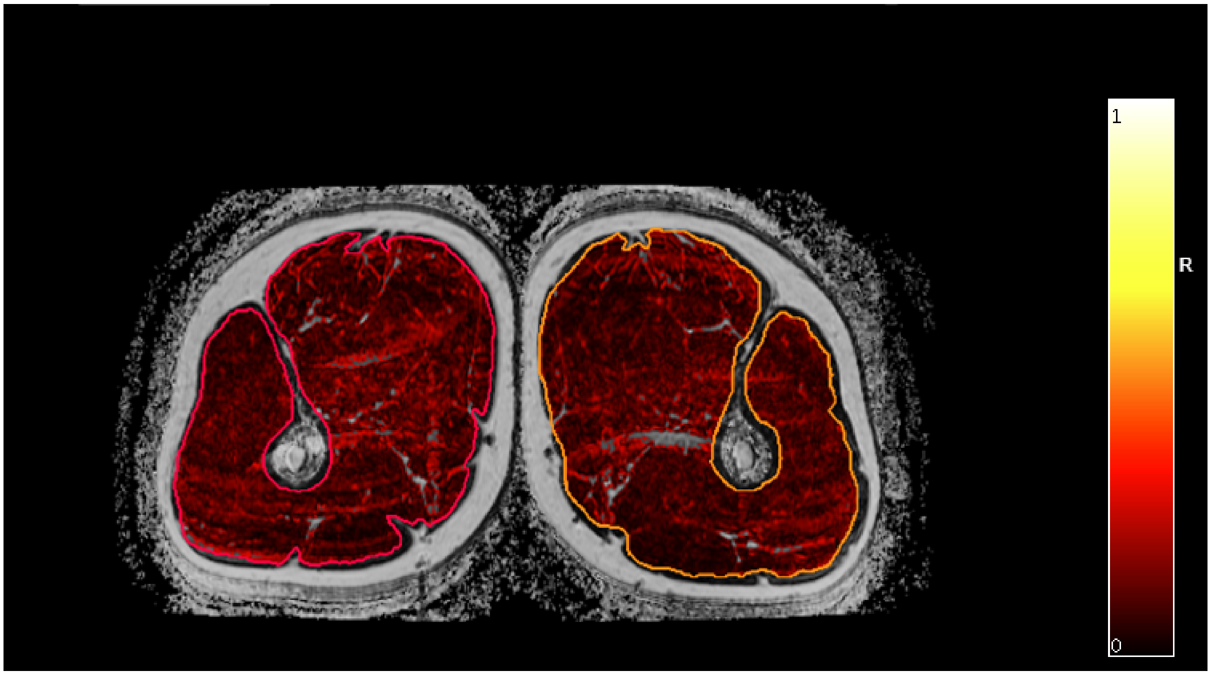

Supplement: Supplementary file 1 [file Data_Sheet_1.PDF]
